# Clinical outcomes among adolescents living with HIV in Kenya following initiation on antiretroviral treatment

**DOI:** 10.1371/journal.pgph.0000094

**Published:** 2022-02-22

**Authors:** Judith Kose, Appolinaire Tiam, Stephen Siamba, Cosima Lenz, Elizabeth Okoth, Theresa Wolters, David van de Vijver, Natella Rakhmanina

**Affiliations:** 1 Elizabeth Glaser Pediatric AIDS Foundation, Nairobi, Kenya; 2 Elizabeth Glaser Pediatric AIDS Foundation, Washington, DC, United States of America; 3 Department of Medical and Scientific Affairs, Washington, DC, United States of America; 4 ErasmusMC, Department of Viroscience, Rotterdam University, Rotterdam, Netherlands; 5 University of Bergen, Centre for International Health, Bergen, Norway; 6 Department of Pediatrics, The George Washington University, School of Medicine and Health Sciences, Washington, DC, United States of America; 7 Children’s National Hospital, Washington, DC, United States of America; Aarhus University: Aarhus Universitet, DENMARK

## Abstract

In Kenya, HIV/AIDS remains a leading cause of morbidity and mortality among adolescents living with HIV (ALHIV). Our study evaluated associations between demographic and healthcare factors and HIV treatment outcomes among ALHIV in care in Kenya. This retrospective cohort study evaluated the clinical outcomes of newly diagnosed ALHIV enrolled in HIV care during January 2017-June 2018 at 32 healthcare facilities in Homabay and Kakamega Counties. Demographic and clinical data were abstracted from patient clinical records and registers during the follow up study period January 2017-through May 2019. ALHIV were stratified by age (10–14 versus 15–19 years). Categorical variables were summarized using descriptive statistics; continuous variables were analyzed using mean values. The latest available treatment and virological outcomes for ALHIV were assessed. 330 ALHIV were included in the study (mean age 15.9 years; 81.8% female, 63.0% receiving HIV care at lower-level healthcare facilities). Most (93.2%) were initiated on ART within 14 days of diagnosis; 91.4% initiated EFV-based regimens. Of those on ART, only 44.6% were active on care at the end of the study period. Of those eligible for viral load testing, 83.9% were tested with 84.4% viral suppression rate. Retention in care was higher at higher-level facilities (67.5%) compared to lower-level facilities (28.6%). Factors associated with higher retention in care were school attendance (aRR = 1.453), receipt of disclosure support (aRR = 13.315), and receiving care at a high-level health facility (aRR = 0.751). Factors associated with viral suppression included older age (15–19 years) (aRR = 1.249) and pre-ART clinical WHO stage I/II (RR = .668). Viral suppression was higher among older ALHIV. Studies are needed to evaluate effective interventions to improve outcomes among ALHIV in Kenya.

## Introduction

In 2020, an estimated 1.7 million adolescents aged 10–19 years lived with HIV globally, 88% of whom live in sub-Saharan Africa [1]. Adolescents are a heterogeneous population with diverse demographic, social, and clinical characteristics. Younger adolescents living with HIV (ALHIV) aged 10–14 years frequently include those who acquired HIV perinatally and are generally more dependent on their caregivers in their HIV care and treatment. Older ALHIV (15–19 years) are generally more independent and assume more responsibility for their own HIV care, which may intersect with subsequent life transitions such as changes in schools, moving away from home, and new relationships with partners [2, 3]. For female ALHIV, significant life changes such as marriage and motherhood often begin during this period [4, 5].

In Kenya, a country with one of the highest HIV burdens worldwide, AIDS remains a leading cause of morbidity and mortality among adolescents [6, 7]. In 2019, Kenya had an estimated 91,634 adolescents living with HIV (ALHIV), and 2,275 deaths among ALHIV [8]. Due to their unique vulnerabilities and multi-faceted transitions, ALHIV are known to experience worse treatment outcomes compared to adults including lower retention in care and viral load suppression (VS) rates [9–11]. Various factors influence these sub-optimal outcomes including poverty, lack of social support, multiple barriers to antiretroviral treatment (ART) adherence, external and internal stigma, and HIV status disclosure challenges [12–15]. Global reported disclosure rates among children and adolescents range between 0–69% with estimates in Kenya as low as 36.6% among ALHIV 10–14 years [16, 17].

Several studies have assessed treatment outcomes of ALHIV in Kenya including engagement in care, retention, VS, and mortality [11, 17–19]. Published studies report the mortality for clients initiated on ART ranging from 3.9% at 12 months to 5% at 19 months; retention in care at 65% at 35 months after diagnosis; VS at 67% for younger adolescents and 68% for older adolescents; and lost to follow up (LTFU) rate after 12 months of ART initiation at 10% for young adolescents and 19.8% for older adolescents [11, 17–19]. Key factors associated with favorable HIV outcomes among ALHIV included disclosure of HIV status, accessing adolescent-friendly services, and being on ART for 6–12 months [17, 20, 21]. Structural factors, like type of health facility, health seeking behaviors and additional social factors, including school attendance, have not been explored in these analyses. Our study aimed to evaluate associations between demographic, social and healthcare factors and HIV treatment outcomes among a cohort of ALHIV newly diagnosed and enrolled in care in Homabay and Kakamega Counties in Kenya.

## Methods

### Study design and setting

This was a retrospective cohort study evaluating available programmatic data for ALHIV from 32 sites in Homabay and Kakamega Counties. ALHIV included in the study cohort were between the ages of 10–19 years who were newly diagnosed with HIV and enrolled into care between January 2017 and June 2018 at select healthcare facilities (HCF). ALHIV in the study were followed for the clinal outcomes through May 2019, at which point we retrospectively abstracted programmatic data from their clinical records.

Healthcare data included type of HCF, e.g., dispensaries/health centers, sub county hospitals and county referral hospitals. Site composition included 2 county health referral hospitals (level 5), 7 sub-county/district hospitals (level 4), 13 dispensaries and 10 health centers (levels 2 and 3). HCFs included in the study offered specific adolescent days on a monthly basis that catered to ALHIV, but did not have a permanent space in the clinic dedicated to adolescents.

For this study, sub-county/district and county referral hospitals were categorized as higher-level facilities, with dispensaries and health centers categorized as lower-level facilities. The dispensaries and health centers provide mainly outpatient services. Level 1, community services, were not included in this study. The sub-county hospitals are referral hospitals providing both in-patient and outpatient services, but not specialized services. County referral hospitals provide inpatient and outpatient services, specialized services and serve as the referral facilities for the regions. All sites offer free HIV care and treatment services [22].

### Data collection and statistical analysis

The cohort of ALHIV diagnosed with HIV between January 2017 and June 2018 were identified by reviewing and abstracting HIV testing data from the HTS and referral register including the date of positive HIV test, date of enrollment into care, and reasons for not being enrolled into care for those not enrolled.

For the outcome measures, the latest available patient data at the last ART pick up date for each client as of May 2019 was abstracted from patient registers and files. We abstracted data from the referral register, pre-ART cohort registers, patients’ record cards (blue/green card stored in patient files), individual tuberculosis intensive case finding (TB ICF) cards, adolescent checklists, disclosure checklists, patient summary sheets, electronic medical records (EMR) and laboratory notebooks. Routine viral load (VL) data, including all available VL data for enrolled ALHIV, were extracted from the facility VL tracking registers.

We collected demographic data including age, sex, school attendance, orphanhood (defined as having lost one or both biological parents documented in records), and marital status at the time of enrollment in care. Clinical characteristics included WHO clinical staging at time of enrolment in HIV care, timing of ART initiation after HIV diagnosis, most recent ART regimen, HIV VL eligibility and VL results. According to the standards of clinical care, eligibility for VL testing was defined as ALHIV who have been on ART for ≥6 months. VL test uptake was defined as the number of eligible ALHIV who had a VL test with a documented test result. VS was defined as a VL <1000 RNA copies/mL of plasma or dried blood sample (DBS) [22].

Kenyan ART guidelines from 2016 and 2018 specify use of antiretroviral drugs for treatment and prevention of HIV according to age-band (<15 and ≥ 15yrs) with the preferred regimen for those 3–14 years as ABC + 3TC + EFV and those ≥ 15 years as TDF + 3TC + DTG8 or TDF + 3TC + EFV [23, 24]. The guidelines also recommend VL monitoring at 6- and 12-months following ART initiation, and thereafter on an annual basis (24 months following ART initiation) [22, 23]. ALHIV in the cohort were eligible for a viral load test if by the time of their last ART pick up as of May 2019, they had been on ARVs for at least 6 months; these criteria therefore exclude ALHIV who did not reach the 6 months timepoint for reasons including being transferred out (TO), lost to follow up (LTFU), or those who died. For ALHIV with repeat VL measurements (those in care >12 months), the most recent VL results to the end of the study period were included in the analysis. In line with national guidelines, transition of ALHIV in care to adult care should occur at or after 19 years of age in addition to attaining certain metrics for adherence and retention in care [22]. Transition to adult care was not included as a factor in the analysis as only a portion of enrolled ALHIV would be preparing or navigating the process as well as many transitioning ALHIV transition to adult settings within their existing facility.

HIV treatment outcomes were defined as being: a) *active in care*—documented to have collected ARVs in the last scheduled clinical visit; b) *lost to follow up* (LTFU)—missed ARV collection for a period of 3 or more months since the last visit; c) *transferred out* (TO)—documented as having transferred out of the ARV enrolment facility to another facility; d) *dead*–documented as deceased. Treatment outcomes were further grouped in two major categories as *being active in care* and *inactive in care* (LTFU, TO and dead). TO was grouped as being inactive in care with the reference point of the facility where the ALHIV transferred out of; this facility would view the transferred ALHIV as being no longer active in care at their facility. The latest available treatment and virological outcomes for ALHIV were assessed.

Data on whether disclosure of HIV status support services were provided were collected as part of HCF characteristics. In the standard of care, enrolled ALHIV receive age appropriate adherence counselling, disclosure support and post-disclosure support services including ongoing counselling [22]. Disclosure counselling is done during routine clinic visits and during ALHIV support group sessions. The data are routinely recorded in the standard HIV treatment patient card (blue/green card) at the facility as part of patient records. The disclosure of HIV status data was extracted from standard clinical records forms. The data collected included documentation of any HIV disclosure related counselling ALHIV had received.

Quantitative data analysis was performed using STATA version 12.0 (College Station, TX, USA). We summarized categorical variables using frequencies and percentages and continuous variables using means (standard deviation (SD)). For the purpose of the analysis, ALHIV were stratified into two age categories: younger ALHIV (10–14 years) and older ALHIV (15–19 years).

We evaluated the association between clinical and demographic characteristics and HIV treatment and VL outcomes using relative risk regression models. The variables included in the relative risk regression models were determined using the Chi-square tests of association between clinical and demographic characteristics and HIV treatment and VL outcomes where factors showing significant association were used in the relative risk regression models. The risk regression model was used to determine the strength of the associations. The binary variable for treatment outcomes was being active in care vs. not being active in care (LTFU, TO and dead) and for viral load outcomes, being virally suppressed vs. being non-virally suppressed. The results in the relative risk regression models comprised the crude/unadjusted and adjusted relative risk at 95% confidence interval (CI). All significant factors were assessed at 5% level of significance.

### Ethical approval

This study was approved by the Kenyatta National Hospital-University of Nairobi Ethics and Research Committee (KNH-UoN ERC)- KNH UoN ERC (P345/04/2016). All data personnel were trained in human subjects research and signed a confidentiality agreement. We obtained a waiver of consent from the KNH-UoN ERC.

## Results

### ALHIV demographic and clinical characteristics

**Fig 1:**
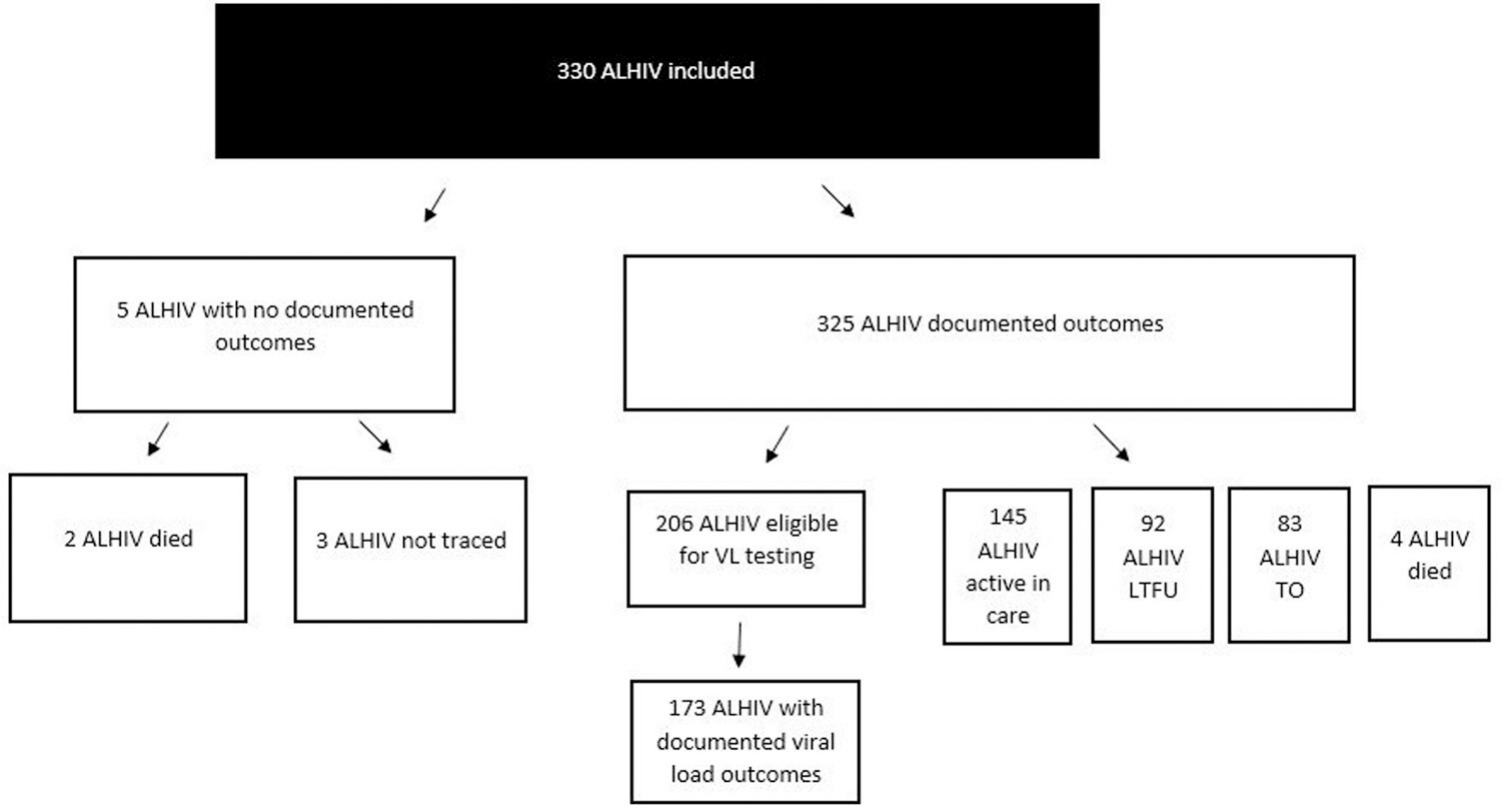
Flow chart of ALHIV included in the study. Active–active in care; LFTU–lost to follow up; TO- transferred out; Dead–died.

We included 330 newly diagnosed ALHIV in the study analysis with a mean age of 15.9 years (SD = 2.6 years); 239 (72.4%) were aged 15–19 years; 270 (81.8%) were females. Less than half of ALHIV (n = 130; 39.4%) were attending school; 33 (10.0%) ALHIV were orphaned and 56 (18.2%) of ALHIV were married, of whom, 55/56 (98.2%) were older ALHIV (Table 1). Out of 330 ALHIV, 325 (98.5%) were enrolled in care; two died before ART initiation and three were never traced back for ART initiation following testing (Fig 1).

**Table 1:** Demographic and clinical characteristics of newly diagnosed ALHIV enrolled in care between January 2017 and June 2018 in select HCFs in Homabay and Kakamega Counties.

ALHIV Characteristics	Levels	Age (years), N = 330		Total
		10–14 years	15–19 years	
		N = 91	N = 239	
**Sex, n = 330**	Male	35 (38.5%%)	25 (10.5%)	60 (18.2%)
	Female	56 (61.5%)	214 (89.5%)	270 (81.8%)
**Attending school, n = 330**	Yes	65 (71.4%)	65 (27.2%)	130 (39.4%)
	No	26 (28.6%)	174 (72.8%)	200 (60.6%)
**Marital status, n = 307** *	Married	1 (1.1%)	55 (25.3%)	56 (18.2%)
	Not married	89 (98.9%)	162 (74.6%)	251(81.8%)
**Orphan hood status, n = 330**	Orphaned	10 (11.0%)	23 (9.6%)	33 (10.0%)
	Not orphaned	81 (89.0%)	216 (90.4%)	297 (90.0%)
**Entry point**	OPD/IPD	62(68.9%)	147(61.5%)	209(63.5%)
	MCH/PMTCT^±^	7(7.8%)	51(21.3%)	58(17.6%)
	CCC/VCT	16(17.8%)	30(12.5%)	46(13.9%)
	TB clinic	2(2.2%)	2(0.8%)	4(1.2%)
	Others	3(3.3%)	9(3.8%)	12(3.6%)
**WHO stage, n = 330**	Stage I/II	85 (93.4%)	231 (96.7%)	316 (95.8%)
	Stage III/IV	6 (6.6%)	8 (3.3%)	14 (4.2%)
**Time to ART initiation from HIV diagnosis in days, n = 325** **	0–14 days	80 (89.9%)	223 (94.5%)	303 (93.2%)
	>14 days	9 (10.1%)	13 (5.5%)	22 (6.8%)
**ART regimen at the time of data collection, n = 325** **	PI-based	3 (3.4%)	0 (0.0%)	3 (0.9%)
	NNRTI-based	78 (86.2%)	16 (6.7%)	302 (92.0%)
	DTG-based	8 (8.9%)	12 (5.1%)	20 (6.2%)
**Treatment outcomes, n = 325** **		**10–14 years**	**15–19 years**	**Total**
		**n = 89**	**n = 236**	
Active		51 (57.3%)	94 (39.8%)	145 (44.6%)
LTFU		18 (20.2%)	74 (31.4%)	92 (28.3%)
TO		19 (21.4%)	64 (27.1%)	83 (25.5%)
Dead		1 (1.1%)	3 (1.3%)	4 (1.2%)
**Viral load (VL) testing eligibility and uptake**	Eligible for VL testing	66 (74.2%)	140 (59.3%)	206 (63.4%)
	VL tests with documented results	58 (87.9%)	115 (82.1%)	173 (83.9%)
**Viral load outcomes, n = 173** ***		**10–14 years**	**15–19 years**	**Total**
		**n = 58**	**n = 115**	
**Suppressed**		42 (72.4%)	104 (90.4%)	146 (84.4%)
Active		36 (85.7%)	79 (75.9%)	115 (78.7%)
LTFU		0 (0.0%)	14 (13.5%)	14 (9.6%)
TO		6 (14.3%)	11 (10.6%)	17 (11.6%)
Dead		0 (0.0%)	0 (0.0%)	0 (0.0%)
**Not Suppressed**		16 (27.6%)	11 (9.6%)	27 (15.6%)
Active		11 (68.8%)	6 (54.5%)	17 (63.0%)
LTFU		2 (12.5%)	4 (36.4%)	6 (22.2%)
TO		3 (18.7%)	1 (9.1%)	4 (14.8%)
Dead		0 (0.0%)	0 (0.0%)	0 (0.0%)

*23 ALHIV did not have documented marital status.

**5 ALHIV were not documented as initiated on ART at the time of data collection.

***132 ALHIV had a repeat VL test at 6 and 12-months; the 12-month results were included in the analysis.

^±^All female.

ALHIV–adolescents living with HIV; WHO–World Health Organization; ART–antiretroviral treatment; EFV- efavirenz; PI–protease inhibitors; NVP–nevirapine; DTG- dolutegravir; LFTU–lost to follow up; TO–transferred out; VL–viral load.

At enrollment into HIV care, 96% of ALHIV (n = 316) were in clinical WHO stage I/II. Among those with WHO clinical stage III/IV, twice as many were younger ALHIV compared to older adolescents (6.6% versus 3.3%). The majority (93.2%) of ALHIV were initiated on ART within 14 days of being diagnosed with HIV (303/325) with a mean number of 5.2 days (SD = 30 days) to ART initiation. The majority (n = 325; 98.5%) of participants had documented treatment outcomes, of whom only 145 (44.6%) were active in care (Table 1). Younger ALHIV were more likely to be active in care compared to older ALHIV (57.3% versus 39.8%, respectively). Ninety-two (28.3%) and 83 (25.5%) participants were LTFU and TO, respectively, and 4 (1.2%) died (Fig 1). Of the 330 ALHIV included in the study, 325 were enrolled on ART. Of the 325, 63% (n = 206) were eligible for VL testing because by the time of their last ART pick up as of May 2019, they had been on ART for at least 6 months. The remaining 119 ALHIV did not meet these criteria as they were either TO, LTFU, or died prior to reaching 6 months on ART. Of the 119 ALHIV, 63 were LTFU, 4 died, and 52 TO. Of the 206 eligible for VL testing, 173 (83.9%) had a VL test with documented results, among whom 146 (84.4%) were virally suppressed (<1000 copies/mm^3^). 76.3% (n = 132) of ALHIV who had conducted a VL test had both a 6 month and 12-month viral load; the 12-month result was included in the analysis. Older ALHIV had higher rates of VS (<1000 copies/mm^3^) compared to younger ALHIV (90.4%. versus 72.4%, respectively).

### ALHIV treatment and viral load outcomes by demographic and clinical characteristics

The age of ALHIV was associated with treatment outcomes (p = 0.005), where 57.3% of 10-14-year-olds were active in care compared to 39.8% of 15-19-year-olds. In addition, 55.2% of male ALHIV were active in care compared to 42.5% of female ALHIV (Table 2). Marital status of ALHIV was not associated with treatment outcomes. The majority of ALHIV (55.1%) who were attending school were active in care compared to 37.9% of those who were not attending school, with school attendance significantly associated with treatment outcomes (p = 0.002). Receipt of disclosure support services was associated with treatment outcomes (p<0.001), where 57.3% of ALHIV who received disclosure support services were active in care compared to 3.9% of ALHIV who did not received disclosure support services. Time to ART initiation and WHO HIV disease stage were not associated with treatment outcomes. Treatment outcomes were also associated with facility type (p<0.001), where 36.1%, 55.0% and 67.5% of ALHIV who received HIV care at dispensaries/health centers, sub-county hospitals and county referral hospitals were active in care, respectively. The proportion of ALHIV who were orphaned and active in care was higher 17/32 (53.1%) compared to 43.7% of ALHIV who were not orphaned and active in care (p = 0.310). Among ALHIV who died, 75% were diagnosed at the stage III/IV and 25% started ART >14 days.

**Table 2:** Factors associated with treatment and viral load outcomes among ALHIV enrolled into HIV care between January 2017 and June 2018 in HCFs in Homabay and Kakamega Counties.

Factor	Level	Treatment outcomes			Viral load outcomes		
		Active in care, N = 145	Not active in care*±±*, N = 180	P-value^*+*^	Suppressed, N = 146	Non-Suppressed, N = 27	P-value^*+*^
Age at diagnosis	10–14 years	51(57.3%)	38(42.7%)	0.005*	42(72.4%)	16(27.6%)	0.002*
	15–19 years	94(39.8%)	142(60.2%)		104(90.4%)	11(9.6%)	
Sex	Male	32(55.2%)	26(44.8%)	0.078	29(82.9%)	6(17.1%)	0.780
	Female	113(42.5%)	153(57.5%)		117(84.8%)	21(15.2%)	
Marital Status***±***	Single	115(46. 6%)	132(53.4%)	0.800	111(82.8%)	23(17.2%)	0.180
	Married	25(44.6%)	31(55.4%)		26(92.9%)	2(7.1%)	
In school	Yes	70(55.1%)	57(44.9%)	0.002*	65(84.4%)	12(15.6%)	0.990
	No	75(37.9%)	123(62.1%)		81(84.4%)	15(15.6%)	
Orphaned	Yes	17(53.1%)	15(46.9%)	0.310	16(72.7%)	6(27.3%)	0.110
	No	128(43.7%)	165(56.3%)		130(86.1%)	21(13.9%)	
Time to ART initiation	0–14 days	134(44.2%)	169(55.8%)	0.600	134(83.8%)	26(16.3%)	0.410
	>14 days	11(50.0%)	11(50.0%)		12(92.3%)	1(7.7%)	
Disclosure support services received	Yes	142(57.3%)	106(42.7%)	<0.001**	142(84%)	27(16%)	0.380
	No	3(3.9%)	74(96.1%)		4(100%)	0(0%)	
WHO stage	Stage I/II	139(44.7%)	172(55.3%)	0.890	142(85.5%)	24(14.5%)	0.043*
	Stage III/IV	6(42.9%)	8(57.1%)		4(57.1%)	3(42.9%)	
Facility Type	Dispensary/Health Centre	74(36.1%)	131(63.9%)	<0.001**	86(86%)	14(14%)	0.690
	Sub-County Hospital	44(55.0%)	36(45.0%)		37(80.4%)	9(19.6%)	
	County Referral Hospital	27(67.5%)	13(32.5%)		23(85.2%)	4(14.8%)	
County	Homabay	116(46.0%)	136(53.9%)	0.340	118(84.9%)	21(15.1%)	0.710
	Kakamega	29(39.7%)	44(60.3%)		28(82.4%)	6(17.6%)	

Level of significance

* <0.05

** at 0.001. **±**22 ALHIV did not have documented marital status. ALHIV–adolescents living with HIV; WHO–World Health Organization; ART–antiretroviral treatment; ±±lost to follow up, transferred out, died;.

^+^ Chi-square test.

The age of ALHIV was associated with VL outcomes; 72.4% and 90.4% of 10–14 and 15-19-year-old ALHIV were suppressed respectively (p = 0.002). The VS rate among male and female ALHIV was 82.9% and 84.8%, respectively, while among married ALHIV, it was 92.9%. In addition, the VS rate was the same among ALHIV attending or not attending school and was 86.1% among non-orphaned ALHIV compared to 72.7% among orphaned ALHIV. The ALHIV who were initiated on ART more than 14 days after their HIV diagnosis had a VS rate of 92.3% compared to those initiated on ART within 14 days who had a VS rate of 83.8%. Eighty four percent (84%) of ALHIV who received disclosure support services were suppressed. A higher percentage (85.5%) of ALHIV with WHO stage I/II had suppressed compared to 57.1% of ALHIV with stage III/IV (p = 0.043). The VS rate was 86%, 80.4% and 85.2% among ALHIV receiving HIV care in dispensary/health centers, sub-county hospitals and county referral hospitals respectively, while the VS rate was 84.9% and 82.4% among ALHIV from Homabay and Kakamega Counties.

Consequently, the following variables were considered for the relative risk regression model; age, school attendance, receipt of disclosure support services and facility type for treatment outcomes and age and WHO staging for VL outcomes (Table 2).

The 15-19-year-old ALHIV were 0.695[0.548–0.882] (adjusted RR: 1.165[1.003–1.354]) times as likely to be active in care compared with 10-14-year-old ALHIV (Table 3). The proportion of ALHIV who were active in care was 57.3% and 39.8% among 10–14 and 15-19-year-olds, respectively.

**Table 3:** Relative risk of treatment (active in care) and VS outcomes in relation to associated factors of ALHIV enrolled in HIV between July 2017 and June 2018 in select HCFs in Homabay and Kakamega Counties.

Factor	Levels	Crude Relative Risk (RR), [95% CI], p-value	Adjusted Relative Risk (RR), [95% CI], p-value
**Treatment outcome, Active in care**			
**Age, years**	10 to 14	Ref(1) ^±^	Ref(1)
	15 to 19	0.695[0.548–0.882], 0.003*	1.165[1.003–1.354], 0.046*
**Attending school**	Yes	1.455[1.147–1.845], 0.002*	1.453[1.221–1.729], <0.001**
	No	Ref(1)	Ref(1)
**Disclosure support services received**	Yes	14.696[4.822–44.790], <0.001**	13.315[4.369–40.575], <0.001**
	No	Ref(1)	Ref(1)
**Facility type**	Dispensary/ Health Center	0.535[0.403–0.709], <0.001**	0.566[0.470–0.681], <0.001**
	Sub-County Hospital	0.815[0.608–1.092], 0.170	0.751[0.628–0.897], 0.002*
	County Referral Hospital	Ref(1)	Ref(1)
**Viral load outcome, VS**			
**Age, years**	10 to 14	Ref(1)	Ref(1)
	15 to 19	1.249[1.054–1.479], 0.010*	1.249[1.054–1.479], 0.010*
**WHO stage**	Stage I/II	Ref(1)	na
	Stage III/IV	0.668[0.351–1.273], 0.220	na

Level of significance

* <0.05

** at 0.001, na- not applicable where unadjusted/crude RR is not significant. RR- Risk Ratio/Relative Risk.

^±^REF–reference.

School attendance was associated with being active in care; 82.4% of ALHIV attending school were active in care compared to 62.9% of ALHIV who were not attending school. The results in Table 3 show that ALHIV attending school were 1.455[1.147–1.845] (adjusted RR: 1.453[1.221–1.729]) times as likely to be active in care as compared to those not attending school.

Furthermore, ALHIV who had received disclosure support services were 14.696[4.822–44.790] (adjusted RR: 13.315[4.369–40.575]) times as likely to be active in care as those who had not received disclosure support services (Table 3). The results in Table 2 also show that receipt of disclosure support services was associated with being active in care where the proportion of ALHIV who received disclosure support services and were active in care was 74.2% compared to 18.2% among ALHIV who did not receive disclosure support services.

The ALHIV who were receiving HIV care services at dispensary/ health centers were 0.535[0.403–0.709] (adjusted RR: 0.566[0.470–0.681]) times as likely to be active in care as ALHIV receiving HIV care services at county referral hospitals. Furthermore, ALHIV receiving HIV care services at sub-county hospitals were 0.815[0.608–1.092] (adjusted RR: 0.751[0.628–0.897]) times as likely to be active in care as those receiving HIV care services at county referral hospitals in the adjusted model (Table 3).

In addition, the WHO stage was associated with treatment outcomes; the proportion of ALHIV active in care was 85.5% and 57.1% among those in stage I/II and stage III/IV, respectively. However, the results in Table 3 indicate that although ALHIV in stage III/IV were 0.668 [0.351–1.273] times as likely to be active in care as those in stage I/II, this association was not significant. Age was associated with virological outcomes, with 15-19-year-old ALHIV being 1.249 [1.054–1.479] times as likely to be virally suppressed compared to 10-14-year-old ALHIV. The proportion of ALHIV with VS was 72.4% and 90.4% among 10–14 and 15-19-year-old ALHIV, respectively.

### ALHIV treatment outcomes by healthcare facility types

**Fig 2:**
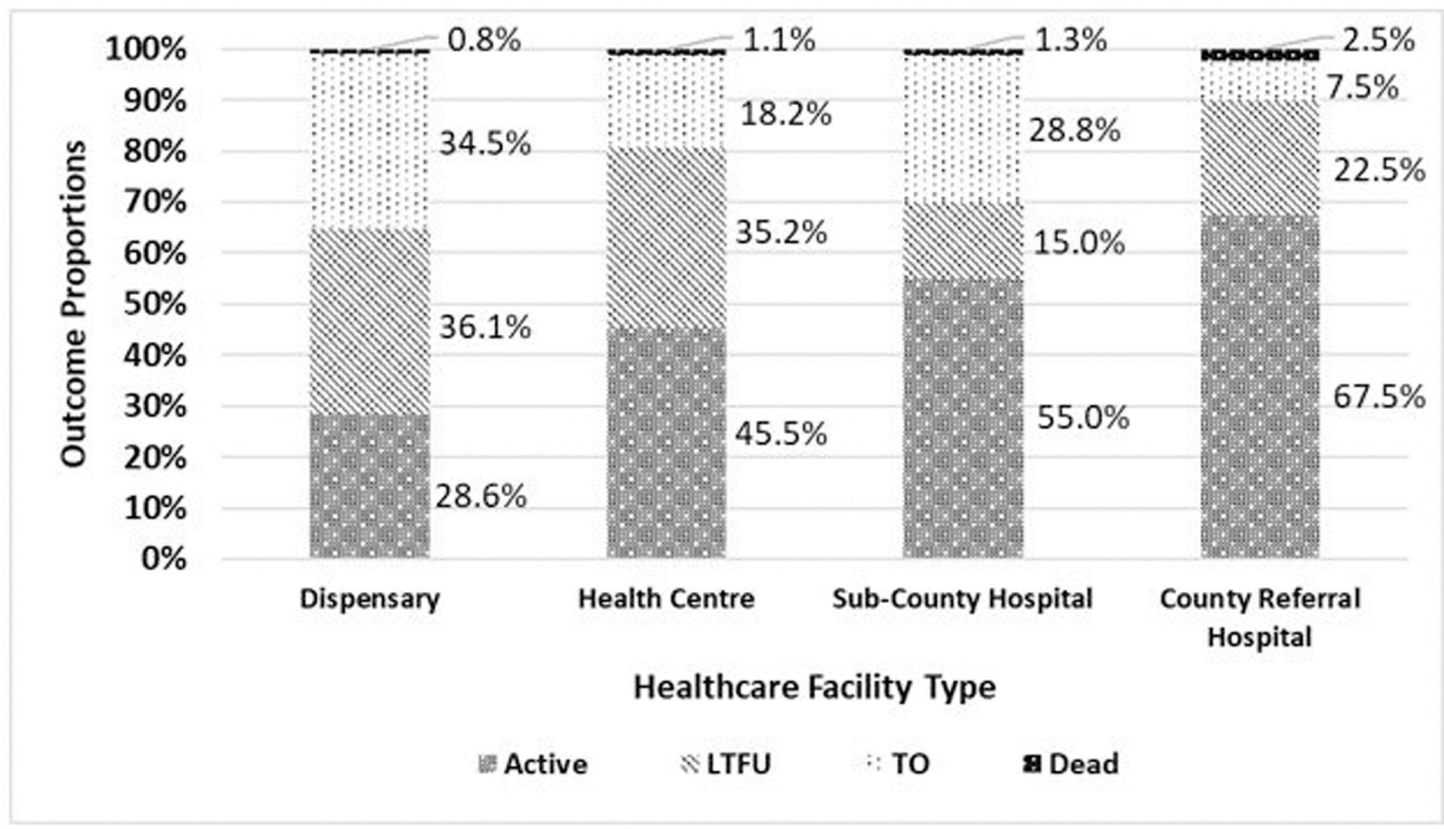
Treatment outcomes by facility type. Active–active in care; LFTU–lost to follow up; TO- transferred out; Dead–died.

The majority (64%) of ALHIV (n = 208) were receiving HIV care at lower-level facilities. Higher-level facilities had the highest proportion of ALHIV active in care with 55.0% at sub-county hospitals and 67.5% at county referral hospitals. The rates of LTFU were highest among lower-level facilities at 36.1% at dispensaries and 35.2% at health centers (Fig 2).

## Discussion

Our study identified important associations between age, school attendance, sex, disclosure support, and receipt of services at various healthcare facility levels with retention in care and treatment outcomes among ALHIV in Kenya. Consistent with other studies from Kenya, most of the ALHIV in our study were diagnosed with HIV before progression to late WHO stages [24] and enrolled in care at lower-level facilities including health centers and dispensaries [25]. This is most likely due to the fact that testing and initial HIV care are widely available at lower-level facilities in Kenya, [26, 27] and adolescents frequently seek services at lower-level clinics in Kenya and South Africa [28, 29].

Suboptimal retention in care among ALHIV is a known barrier to achieving sustained VS and preventing HIV associated morbidity and mortality. Our study highlights the persistent challenge of retaining ALHIV in care with 44.6% of ALHIV active in care during the study period. This finding indicates more than half of ALHIV were LTFU, TO or died. High rates of TO and LTFU among ALHIV in our study can be associated with adolescents being a highly migrant population in sub-Saharan Africa [30–33]. Though not statistically significant, we observed high rates of being active in care among orphaned ALHIV, and speculate that this could be due to orphaned ALHIV receiving support services within the Orphans and Vulnerable Children (OVC) programs that promote their engagement in care. Studies report that receipt of social protection services, similar to those included in OVC packages, may lead to improved HIV treatment outcomes including improved adherence and retention in care among children and ALHIV [34–36]. Support services to orphans also frequently include legal and social assistance, as per the OVC package, which can facilitate retention in HIV care among orphaned ALHIV.

We observed higher retention in care among ALHIV who were male, in school, receiving disclosure support services and attending a higher-level facility. These findings are similar to other studies that found disclosure support to be associated with improved retention outcomes [37–40]. Disclosure has been reported as a facilitator for adherence in reducing stigma from family members in taking treatment and improving receipt of social support [39]. Disclosure of HIV status to adolescents may further present opportunities for increase access to adherence and psychosocial support [41]. Disclosure support from health providers is critical to facilitate disclosure of HIV status to adolescents alongside other stakeholders including caregivers [40].

Despite age not being a significant factor concerning retention in care, our study did show younger ALHIV were less likely to be virally suppressed compared to their older peers. Similarly, a study in Kenya found young adolescents to have lower VS compared to older adolescents and young adults [19]. There were also fewer young adolescents in this study on DTG-based regimens compared to older adolescents; however, the numbers were small. This lower VS among young adolescents could stem from a significant proportion of ALHIV in the study having been perinatally infected and thereby experiencing a longer duration of the disease, suboptimal past treatment regimes, potentially more compromised immune system as well as behavioral challenges with adherence such as low treatment self-efficacy, limitations of caregiver treatment literacy, including not understanding side effects, or not being able to attend care appointments due to competing priorities (e.g., like attending school) [42–46].

Though not statistically significant, our results revealed high retention in care among ALHIV who were male in our predominately female ALHIV cohort. The literature on retention shows mixed results concerning differences by sex. Similar to findings in our study showing higher retention among male ALHIV, a study from Uganda reported being male was independently associated with retention in HIV care [47]. Moreover, a study of prevalence and predictors of retention in care among adolescents in South Africa found male adolescents to be significantly more likely to be retained in care compared to females [48]. Lower retention among female ALHIV could stem from a variety of factors including stigma and discrimination, competing priorities in the home, and pregnancy [49]. Several other studies have highlighted worse retention rates among HIV positive adolescent girls who are pregnant [50, 51]. However, some studies have demonstrated higher retention in care rates among females compared to males. A study from Mozambique reported that female ALHIV (including pregnant and breastfeeding women) were more likely to be retained in care compared to males in the same age cohort [52]. Furthermore, a study from Tanzania reported consistently higher retention in care among females compared to males through a 36-month follow-up period [53]. It is possible that these differences in gender-based HIV treatment and care outcomes are determined by other socioeconomical and cultural factors within diverse national settings.

Contrary to other studies conducted in Kenya that have reported school attendance and associated stigma as barriers to retention in HIV care, in our study, school attendance was associated with higher retention in care among ALHIV [54, 55]. During the study period, ongoing support for school-going adolescents, including the PEPFAR-funded DREAMS program, in Kenya may have contributed to ALHIV staying in care [56–58]. In our study, the majority of ALHIV attending schools were younger (10–14 years) compared to those not in school, which would explain their higher retention in care. A positive, non-stigmatizing environment at school can potentially play a critical role in supporting ALHIV to stay active in care, as shown in the Red Carpet program providing boarding school-based ALHIV support in Kenya [59].

In our study, higher-level facilities (sub-county/county referral hospitals) had a significantly higher proportion of ALHIV active in care, compared to the lower-level facilities (dispensaries/health centers) that had a higher proportion of LTFU among adolescent patients. The quality of HIV services often differs by level of facility, with higher-level facilities having capacity to provide more comprehensive multidisciplinary HIV treatment and support services. There is mixed evidence in the literature, [60] with some evaluations highlighting high rates of LTFU at higher-level facilities compared to lower-level facilities, [61–64] and other studies identifying higher LTFU at lower-level facilities [65, 66]. This may be due to the fact that higher-level facilities are often equipped with better infrastructure including staffing, availability of HIV expertise and relevant support services, and offer comprehensive care including peer support groups, enhanced counselling, and stronger defaulter tracing activities compared to lower-level facilities. Higher death rates observed in this study at higher-level facilities may have resulted from patients with more severe disease and opportunistic infections being referred or attending higher-level facilities [67]. In lower-level facility settings, worse retention could be attributed to high patient-to-provider ratios limiting individualized patient education, insufficient support for ART management, and limited ART counselling capacity [68–70].

In our study, we observed higher VL test uptake among ALHIV (83.9%) compared to other published studies of ALHIV in Kenya ranging from 75%-78.6% in Machakos and 75% in Western Kenya [18, 71]. Overall, the VS rate (84.4%) in our study of ALHIV on predominantly EFV-based ART was also significantly higher compared to the national VS rate of 61.4% among ALHIV (10–19 years of age) [6]. Similar to another study in Kenya, older adolescents (15–19 years) had higher VS rates in our cohort compared to younger adolescents (10–14 years) [19]. While the VS rates were high, there is a need for longitudinal follow-up, as a recent study on ALHIV in Kenya demonstrated that high VS rates of 74% was not sustained overtime, decreasing to 52% after 38-month follow-up period [18].

Similar to our findings of higher VS rates among ALHIV with WHO stages I/II compared to those in WHO stages III/IV, another study from Kenya reported diagnosis of WHO stage I to be associated with higher rates of VS [72]. Comparably, a study evaluating the HIV continuum of care among adolescents and young adults 12–24 years in the United States, found that newly diagnosed patients with advanced disease were more likely to have higher VLs.[73] A study from Eswatini found that those pediatric, adolescent and adult patients with WHO stage III and IV HIV disease were more likely to have a detectable VL [74].

Our study has several limitations. We conducted a retrospective cohort study that relied on the accuracy and completeness of the standard of care patient records collected by facility staff. Furthermore, we collected data on the availability of disclosure support, but did not have data on the disclosure status of participants. ALHIV in our study were predominantly female (82%), which could have impacted our findings on gender differences and overall outcomes. Pregnancy status among female participants at the time of HIV testing, ART initiation and throughout follow-up was not collected and therefore analysis in relation to pregnancy was not conducted. While we report higher rates of retention among orphaned ALHIV, we did not have data on any additional support services these ALHIV may have received from the OVC programs. Our study analyzed the data on only newly enrolled ALHIV and, therefore, the findings are limited to this group; however, our results may be less biased than studies that enroll all ALHIV, including those identified in childhood who have been retained in care. Nevertheless, we were able to collect patient level data on the outcomes of HIV within a large cohort of ALHIV from an area of high HIV prevalence in Kenya and identified several important associations with retention in care and VS in this vulnerable population.

## Conclusions

Our study demonstrates successful linkage to ART among the majority of newly identified ALHIV in two counties in Kenya. The majority of ALHIV received care in lower-level healthcare facilities in Kenya, yet higher-level facilities had higher retention in care rates, demonstrating the need for additional evaluation of strategies to optimize ALHIV care at lower-level facilities. Despite prompt ART initiation, there was a low rate of retention in care in the cohort. Retention in care was associated with school attendance and access to disclosure support, and receiving care at a high-level facility. Among ALHIV in care with a documented VL result, the majority were virally suppressed. Attending school was associated with higher retention in care, while being older (15–19 years) was associated with having higher odds of being virally suppressed. Further studies are needed to better understand the associated factors and develop effective interventions to increase retention in care and improve outcomes among ALHIV in sub-Saharan Africa.
